# Trajectory to local extinction of an isolated dugong population near Okinawa Island, Japan

**DOI:** 10.1038/s41598-022-09992-2

**Published:** 2022-04-12

**Authors:** Hajime Kayanne, Takeshi Hara, Nobuaki Arai, Hiroya Yamano, Hiroyuki Matsuda

**Affiliations:** 1 Department of Earth and Planetary Science, The University of Tokyo, Hongo, Tokyo 113-0033 Japan; 2 Japan Fisheries Science and Technology Association, Akasaka, Tokyo 107-0052 Japan; 3 National Fisheries University, Shimonoseki, Yamaguchi 759-6595 Japan; 4 Biodiversity Division, National Institute for Environmental Studies, Tsukuba, Ibaraki 305-8506 Japan; 5 Faculty of Environment and Information Sciences, Yokohama National University, Yokohama, 240-8501 Japan

**Keywords:** Conservation biology, Ecology, Population dynamics

## Abstract

A small animal population becomes extinct owing to demographic and environmental stochasticity after declining below the minimum viable population (MVP). However, the actual process of extinction derived by stochastic factors after crossing MVP has not been recorded for long-lived marine mammals. Here, we reconstructed the declining history of a small, isolated population of dugongs in Okinawa over 125 years. The initial population size of approximately 280–420 in the nineteenth century declined to approximately < 100 in 1917 because of overfishing, < 70 in 1979, 11 in 1997, 3 after 2006, and all known individuals disappeared or died by 2019. After 1979, a decline in the natural growth rate has led to extinction. Long-lived animals may persist for a few decades after the population falls below the MVP, at which time active conservation measures, such as captive breeding, should be implemented.

## Introduction

Sustainability and extinction of a small animal population is determined by extrinsic (e.g., hunting and habitat loss) and intrinsic (e.g., stochastic reproduction and mortality) factors^1,2^. In general, human-induced extrinsic factors first reduce the population size to a minimum viable population (MVP); then, demographic and environmental stochasticity, along with human activities, lead to extinction. Extinction probability is estimated by population viability analysis and the MVP is often estimated as approximately 50 (ref.^3^), but should change depending on the species, age structure, and genetic composition of a population, and should, therefore, be determined for different species and populations. However, the actual history of a population decrease to extinction with causal factors has not yet been recorded, particularly for long-lived marine mammals; such study requires a long observation period, although many of these species are at the edge of extinction. From this perspective, effective measures to avoid species extinction should be taken based on the practical number of MVP along with possible extinction drivers. In this study, we reconstruct the declining history of a small, isolated population of dugongs in Okinawa for over 125 years.

The dugong (*Dugong dugon*) is a marine mammal inhabiting tropical seagrass meadows for feeding grounds. It is a flagship species for tropical marine ecosystems and is currently classified as ‘vulnerable’ on the IUCN Red List globally^4^. As the population has declined because of hunting, coastal development, and habitat destruction, effective conservation measures need to be addressed^5^. The average natural life span of dugongs is not known; the oldest specimen is suggested to be 73 years old, and only one-tenth of the population is 36 years old or more^6–8^. Dugongs mature at a minimum of 7 years of age and give birth to calves with an interval of three to seven years. The population growth rate is suggested to be 5%, corresponding to the life history presented in this study.

**Figure 1:**
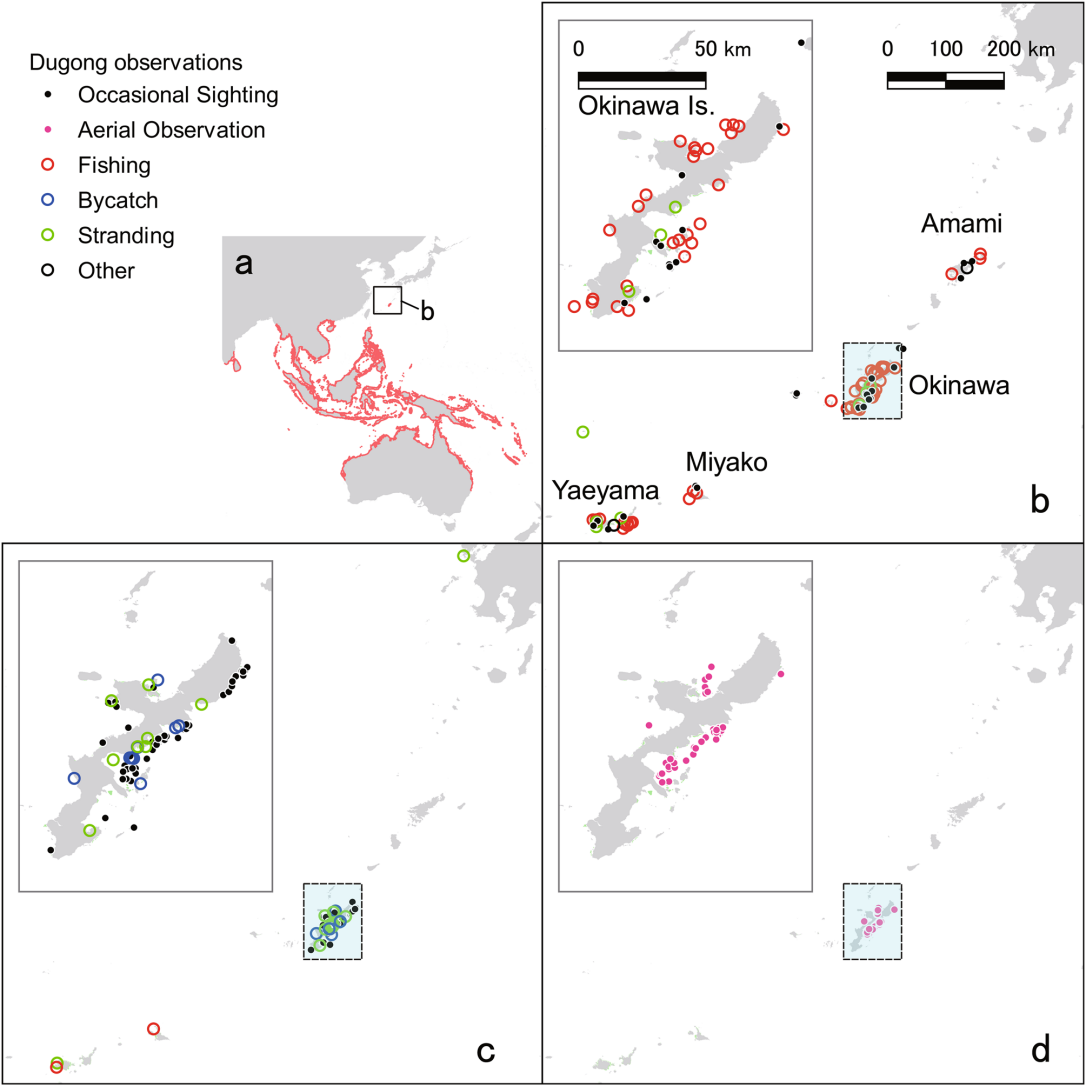
Observation and/or hunting records of dugongs. (**a**) Distribution of dugongs in the southeast Asia and Australia (red). (**b**) Historical dugong hunting and observation locations near the Ryukyu Islands before 1965. (**c**) Occasional sightings and records of bycatch and stranding (dead body drifted ashore) since 1965. (**d**) Aerial observation since 1997. The data from different observation are indicated by different-colored circles. The data were compiled from (Refs^4,14,25–29^ and Supplementary Table 1). Maps were created by QGIS, ver. 3.10.6, https://www.qgis.org/ja/site/forusers/download.html.

The largest known population of dugongs is located in the coastal waters of northern Australia, comprising almost 70,000 individuals^8^. In southeast Asia, dugongs inhabit coastal waters near Indonesia, Malaysia, and Thailand, and a few hundred to a thousand individuals live in each country (Fig. 1a). In the Philippines, dugong populations are fragmented and, in Taiwan, they have become locally extinct^9^. A small population of dugongs had been found since the late 20th century in the coastal waters of Okinawa Island, part of the Ryukyu Islands of Japan—in the northern part of the dugong distribution in the western Pacific—^10^. The dugongs in Okinawa are a geographically distinct group with little demographic or genetic exchange with other populations, and is defined as a subpopulation according to the IUCN Red List^11^.

In 2019, the IUCN designated the dugong subpopulation in the Nansei Islands (Ryukyu Islands) as ‘Critically Endangered’ with a very low number of individuals and the current population trend as ‘Decreasing’^11^, and the Japanese Ministry of the Environment also designated the dugongs in Japan as ‘Critically Endangered’ in 2020. The total subpopulation in 2019 was estimated to number less than 10 animals, according to aerial surveys conducted during 1998–1999^12^. The IUCN assessment report stated that neither a scientifically valid estimate of its size nor the actual trend of the decrease is available^11^. We reconstructed the change in the most probable number of dugong individuals for the last 125 years from fishery statistics, local newspaper articles, aerial observations, and population dynamics models.

## Results

The Okinawa dugong has been reported since, at least, the seventeenth century^13^. From the 17th to nineteenth centuries, dugongs were hunted as a tax to the Ryukyu Dynasty in the Yaeyama Islands—the southernmost islands in the Ryukyus—in a period when hunting was sustainable and maintained the dugong population (Fig. 1b). However, after the Ryukyu Dynasty collapsed and was integrated into Japan in 1879, dugongs began to be harvested commercially.

**Figure 2:**
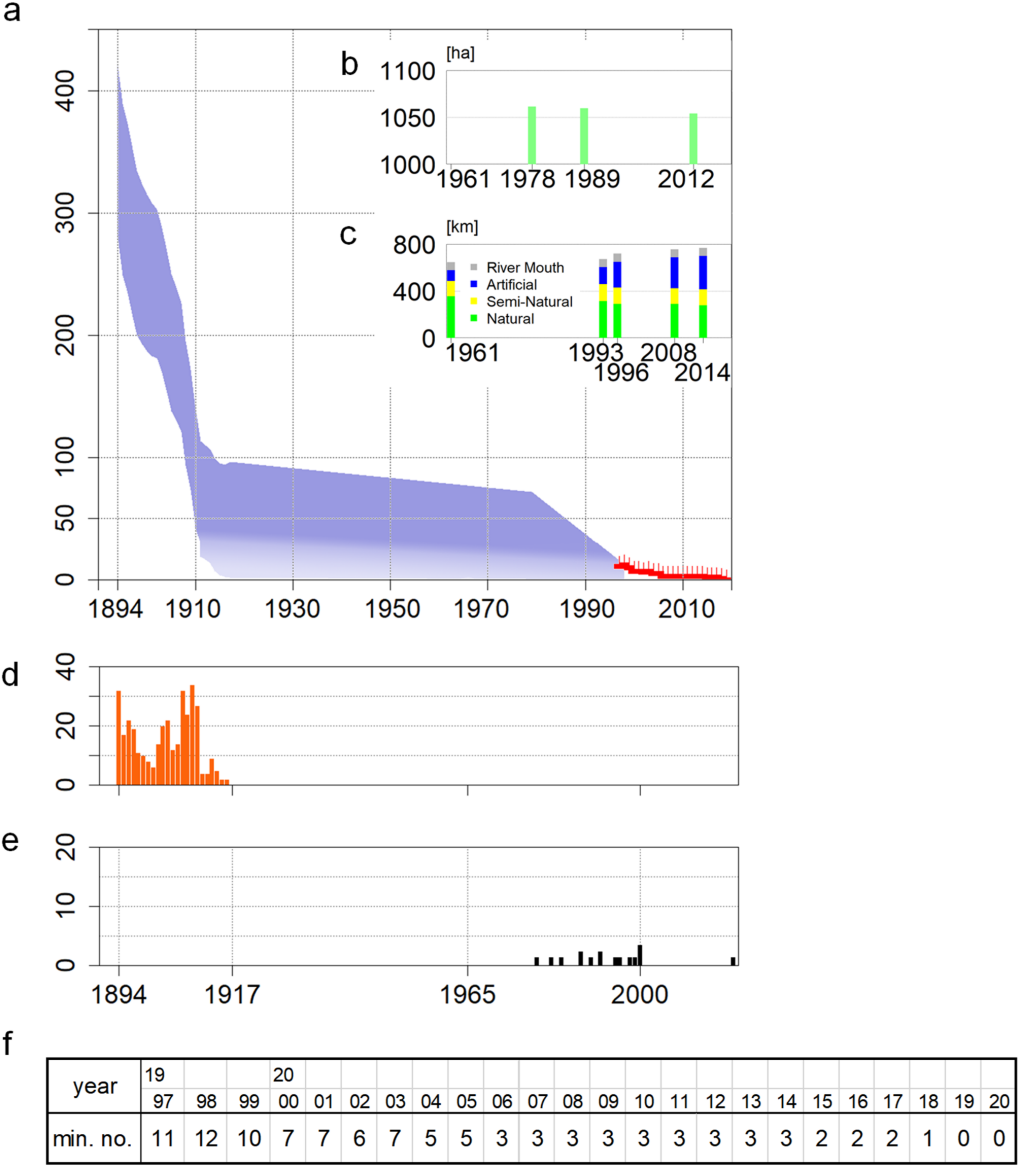
Time-series changes in the number of individual dugongs from 1894 to 2020. (**a**) The estimated number of dugongs in coastal waters near the Ryukyu Islands. (**b**) Change in the area of seagrass around Okinawa Island (Extended Data Fig. 2). (**c**) Lengths of natural (green), semi-natural (yellow), and artificial (blue) coastlines around Okinawa Island in 1961, 1993, 1996, 2008, and 2014 (Extended Data Fig. 3). (**d**) Number of dugongs captured per year from 1894 to 1916 (Extended Data Fig. 1) (refs.^14,15^). (**e**) Records of dugong stranding or caught as bycatch since 1979. (**f**) Minimum number of dugongs identified by aerial observation and bycatch/stranding records since 1997 (Supplementary Data).

The harvest statistics from 1893 to 1916 showed that more than 300 dugongs were caught during this period near the Yaeyama, Miyako, and Okinawa Islands (Fig. 2d, Extended Data Fig. 1a, b)^13–15^. However, from 1911 onwards, the number of captures decreased, and the last reported individual captured was a calf near Okinawa in 1916, suggesting that hunting pressure resulted in a decreased population size. To reconstruct the decline of the dugong population in these islands, we applied a deterministic logistic model (see Methods) and estimated the population size in 1894 and 1917 based on the number of annual catches (*C*_*t*_) and natural population growth rate (λ), ranging from 1 to 1.05 per year. We assumed that the earlier population (*N*_1893_) reached the carrying capacity, and that the population in 1917 (*N*_1917_) was significantly reduced by capture, but persisted from 1894 to 1916. Therefore, *N*_1894_ must be > 329 and > 280 if λ = 1 and 1.05, respectively. In addition, as the intensive catch during 1907–1910 lead to a marked decline in the catch after 1911 (Fig. 2d), which suggested a remarkable reduction in the population size during this period (such as 50%), *N*_1894_ must be < 420 and < 347 if λ = 1 and 1.05, respectively, and the population size in 1917 must be < 96 (Extended Data Fig. 1a). Therefore, the estimated population sizes for 1894 and 1917 were 280–420 and approximately < 100, respectively (Fig. 2a).

No record of dugongs is available regarding 1917 to 1959; however, mortality was inferred to have increased because of the use of explosives (dynamite) to harvest fish and dugongs^15^. Even though dynamite fishing was prohibited after 1948, such fishing continued until 1972 (ref.^16^), when Okinawa Prefecture returned to Japan from US occupancy, and the dugong was designated as a natural monument of Japan. Since 1960, reports of hunting, bycatch, stranding, and dead bodies drifting ashore have been reported in local newspaper articles (Fig. 1c; Supplementary Table 1). Near Amami, Yaeyama and Miyako Islands, four dugongs were reported as hunted for commerce in 1960, 1965 and 1967 (N1, N2 and N3 in Supplementary Table 1), which reveals that dugong had been a fishery target at least by that time. In 1987, a dead specimen was found on Yaeyama shore, and no dugongs were observed on these islands—the Okinawa dugongs have been isolated.

From 1979 to 2004, a local newspaper reported that 14 dugongs were found dead on Okinawa Island: seven were caught in gill or fixed nets (another four were released alive or bred in an aquarium), and seven dead bodies drifted ashore (Fig. 2e, Supplementary Table 1, Supplementary Data). Another dugong (N22 in Supplementary Table 1) was found dead on shore at Kumamoto, 650 km north of Okinawa, from where it might have strayed^17^. Six dead individuals out of the 14 were calves 2 months to several years old. Notably, a pregnant female was caught in a fixed net in 1995, which shows that the Okinawa dugongs reproduced until 2002, given the existence of the calves. Bycatch continued until at least 2000 (N21), but no bycatch deaths have been reported since then.

**Figure 3:**
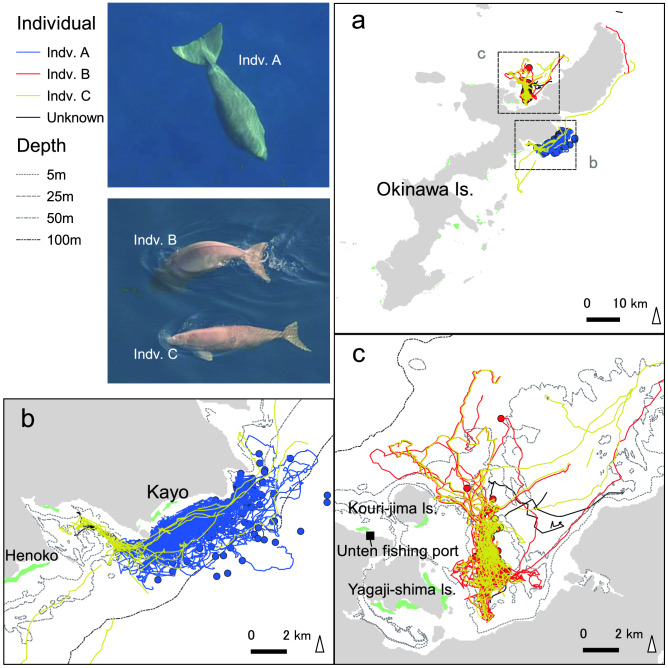
Tracks of the three dugongs (A, B, and C) identified since 2007 (ref.^30,31^). (**a**) Tracks around Okinawa Island. (**b**) Tracks on the coast of Kayo. (**c**) Tracks on the eastern side of Kouri-jima and Yagaji-shima Islands. Maps were created by QGIS, ver. 3.10.6, https://www.qgis.org/ja/site/forusers/download.html.

Since 1997, aerial observations of the Okinawa dugongs have been intensively conducted, and 15 dugongs have been identified (Fig. 1d). Though dugong individuals had not been identified during the year before 2006, we collated the most probable number of individuals observed and found dead during the year by area, and counted the minimum number of the individuals since 1997 (Fig. 2f, Supplementary Data). In 1997, the most probable number of dugongs was 11. In 1998, a two-month-old calf was caught in a gill net and died, indicating that the number temporarily reached 12 (N17 in Supplementary Table 1). From 1999 to 2002, four dead bodies drifted ashore, and one dugong was caught dead in a gill net. Another four have not been observed since 2004 and 2006. Since 2007, only three individuals (hereafter referred as A, B, and C) have been identified, along the eastern and northwestern coasts (Fig. 3). Aerial surveys have covered dugong habitats all around Okinawa Island once a month from 2007 to 2009, and four times a year since 2009 (Extended Data Fig. 4).

Dugong A was male and its observed tracks were restricted to 3 km offshore Kayo (Fig. 3b). Feeding trails had also been observed constantly in a seagrass meadow on a reef flat of Kayo. On the other hand, main habitat of dugongs B (female) and C (sex unknown) was identified in a bay between the main Okinawa Island and Kouri-jima Island (Fig. 3a). They were frequently observed swimming side by side suggesting dugong C was a calf of dugong B (Fig. 3). Dugong C sometimes migrated to the eastern side of Okinawa Island (in one case accompanied by dugong B), and was finally observed in June 2015. Dugong C might have migrated to a remote location, possibly to search for its own habitat or mate. Dugong A has not been observed since September 2018, and the feeding trails have not been observed since then. Dugong B was found dead, stabbed by a ray barb, and drifted ashore at Unten fishing port, on the south coast of Kouri-jima Island on 18 March 2019 (Extended Data Fig. 6).

The population size in 1979 (*N*_1979_) and growth rate (λ) were estimated by approximate Bayesian calculation based on bycatch records from 1979 to 2019, constrained by the number of individuals since 1997. If we ignore stochasticity, *N*_1997_ and *N*_2019_ are represented by *N*_1979_λ^18^ and *N*_1979_λ^40^, respectively. The median and 95% credible interval of *N*_1979_*λ*^40^ for the adopted trials are shown in Extended Data Fig. 7a. When *λ* ≥ 1.005, the reported bycatch mortality was unlikely to satisfy the numbers of *N*_1997_ ≥ 11 and *N*_2019_ ≤ 1. Even if *N*_1979_ > 100 and λ < 0.975, there would be over 11 individuals in *N*_1997_ and possible extinction in 2019. When λ > 0.96, less than 1% of the trials had *N*_1997_ ≥ 72, suggesting reductions in *N*_1979_ < 70 and λ, likely below 1 (Fig. 2a).

## Discussion

The reduction in the Okinawa dugong subpopulation is attributed to extrinsic factors such as hunting, habitat loss, and bycatch and to intrinsic factors such as demographic stochasticity. Overfishing was the first major driving force for the sharp decline of the Okinawa dugong from 280 to 420 individuals, during the early twentieth century, to the estimated < 70 individuals in the late twentieth century.

Dugong habitats have also been lost because of coastal development, such as reclamation and port construction during this period. The area of seagrass bed was estimated as 10,574 km^2^ in 1979 to 10,554 km^2^ in 1989 and 10,497 km^2^ in 2012 accompanying with decrease in natural coastline (Fig. 2b, c and Extended Data Figs. 2, 3). The seagrass bed area in 1979 provided the lowest environmental carrying capacity for dugongs of < 70 at that time. The reduced area in 2012 was still sufficient for the last three specimens in 2012. Reduction in the quality of the habitat could have affected the negative growth rate, but data to verify the same are unavailable. Thus, we can tentatively conclude that the declining carrying capacity represented by the seagrass bed area was not the major driving factor for the population reduction since 1979.

We examined the impact of bycatch on the population decline between 1979 and 1997 using Population Viability Analysis (see Methods). From *N*_1979_, using the adopted parameter sets with λ > 0.96 shown in Extended Data Fig. 7 we calculated the extinction risk until 2019 with and without bycatch. The average and standard deviation of the extinction risk with and without bycatch were 10.7 ± 12.5% and 10.6 ± 12.3%, respectively, with no significant difference. Anthropogenic factors, such as the release of dugongs after bycatch or boat strike, which have not been reported previously may have caused the mortalities of the dugongs drifted ashore^8^. On assuming that all the reported mortalities were due to anthropogenic factors, the average and standard deviation of the extinction risk with and without anthropogenic mortalities were calculated as 21.6 ± 19.4% and 10.6 ± 12.7%, respectively (*P* = 6 × 10^−5^; Extended Data Fig. 8). Stranding and bycatch mortality have been avoided through some measures since 1979, and thus, the extinction risk could have been halved. However, the assumption that all the dugong deaths are due to anthropogenic activities is the largest estimate of the human impact, considering the last one that dugong B died because of natural causes.

Even if we assume all the reported deaths were due to anthropogenic impacts, positive natural growth rate (or λ > 1) was again unlikely (0.3%) among the adopted trials. Thus, the reduction of population since 1979 was not caused by extrinsic factors of declining carrying capacity and bycatch, but was more likely caused by the intrinsic factor of the reduction in the natural growth rate, which may be derived from genetic deterioration or the Allee effect, or other unknown factors associated with reduced population density.

Only three dugongs had been identified since 2006. However, dugong B was found dead in 2019, and was regarded as the last known dugong in the waters of Okinawa^18^. We are not certain that dugongs A and C are dead; however, dugongs A and C had been observed once to several times every year since 1998 and 2005, but have not been observed since 2018 and 2015, respectively (Supplementary Data). Unidentified individuals may still be present, as possible dugong calls were recorded near Henoko in 2020 by the Okinawa Defense Bureau in 2020 (ref.^19^), feeding traits were observed near Kouri-jima Island in 2020 by the Ministry of the Environment^20^ and Okinawa Prefecture^21^, and occasional dugong sightings by fishermen, divers, or from the land have been reported^18^. However, intensive aerial surveys have not observed any other dugongs in this area since 2006. Sea turtles have been found throughout the area during the same surveys (Extended Data Fig. 5), and if other dugongs were present, they should have been found along with the sea turtles. Based on the aerial observation, it is most likely that only three identified dugongs have been present since 2006, which had disappeared by 2019.

Another possibility is that dugongs exist at islands other than Okinawa, which is also suggested by the possible feeding signs observed at several islands 350–500 km southwest of Okinawa Island^21^. Given that dugongs are able to make long-distance migrations (e.g., 560–1000 km)^22,23^, a more extensive search of a broader geographical area should be required to determine whether dugongs A and C (in particular the latter), or other unidentified ones still alive^18^. In any case, it is difficult to maintain the population with the remaining two dugongs, so we have to say that the Okinawa dugong is near extinct or extinct.

This study first reconstructed and observed the trajectory to possible extinction of the dugong subpopulation, with the number of dugongs declining from < 70 in 1979 to 11 in 1997, 3 in 2006, and finally going almost extinct in 2019. This unfortunate record provides lessons for other endangered animals. First, the critical MVP was assigned as 11 for the Okinawa dugong subpopulation, based on the minimum number count in 1997. Although different in their life cycle parameters, a small subpopulation of Hawaiian monk seals experienced a decrease in the number of adults to 30 and then an increase to 100 within 10 years^24^. These two examples lead to a tentative threshold MVP of 10–30 for marine mammals, below which effective conservation measures should be undertaken.

Second, as the number decreased below the MVP, intrinsic factors drove the reduction and extinction. The population growth rate had reduced since 1979, well before the population crossed the critical extinction risk threshold in 1997. Thus, implementing conventional conservation measures alone to remove extrinsic factors, such as strict prevention of bycatch, and habitat conservation and rehabilitation, though essential for conservation, is insufficient to sustain a small population at the edge of extinction. Active interventions, such as the introduction of dugongs from other subpopulations or captive breeding, should be undertaken.

Third, the possible extinction of dugongs occurred 20 years after the number crossed below the MVP, and at least 40 years after the population growth rate decreased. Dugongs A and B must have been > 20 years old, because we identified them as adults in 2007. Given their long life cycle, the final extinction lags behind the MVP. We should not miss the time at which not only passive, but also active conservation measures should be undertaken to sustain a small population of marine mammals. Moreover, scientifically valid estimates and observations would provide a basis for conservation practice.

## Methods

### Deterministic logistic model

The following population dynamics model was applied to reconstruct the initial dugong population size in 1894 from fishery statistics between 1894 and 1914:

$$N_{t + 1} = N_{t} \left( {1 \, + r{-}r \, N_{t} /K} \right) - C_{t} ,$$

where *r* is the intrinsic rate of population increase, *N*_*t*_ is the population size in year *t*, *K* is the carrying capacity, and *C*_*t*_ is the number of individuals removed from the waters near the Ryukyu Islands in year *t*. The carrying capacity (*K*) in 1893 was sufficient to sustain the initial population of dugongs at that time (*N*_1894_). The intrinsic rate of population increase (*r*) was given between 1 and 5% within a range of natural one.

### Approximate Bayesian calculation

We conducted approximate Bayesian calculation (ABC)^32^ to estimate the number of individuals in 1979 based on bycatch data between 1979 and 2019, and the constraints of the numbers of individuals were 11 in 1997, three in 2007, and almost extinct in 2019. We denoted fecundity as *f*, the survival rate until 1 year old as *s*_0_, the annual survival rate after 1 year old as *s*, the age at maturity as *a*_*m*_, and the physiological longevity as *A.* We assumed that the sex ratio at birth was 1:1 on average; the age at maturity *a*_*m*_ was eight years of age^33^, and the physiological longevity *A* was 73 years^6^. We ignored environmental stochasticity because no mass deaths caused by infectious diseases or changes in survival or mortality rates due to environmental fluctuations have not been recorded during this period. We also ignored density effects because the carrying capacity of the location was sufficiently greater than the initial population size, and our goal was to investigate the possibility of population recovery after a decrease in population using a population dynamics model and estimate the natural growth rate during this period. The detailed extinction risk depends on age structure.

According to the life history parameters, except the physiological longevity compiled by (ref.^33^), the annual survival probability of an *a* year-old individual is *s* for *a* = 1, 2, …, 72; *s*_0_ for *a* = 0, and 0 for *a* = 73; the reproductive probability of an adult female > 8 years old is 2*f*. As the number of years for a population to become extinct or recover depends on age composition, age-specific survival, and reproductive rates, we obtain the population growth rate by the maximum eigenvalue of the following Leslie matrix, **L** = {*L*_*ij*_} (*i* = 1,…73, *j* = 1,…,73) as:

$$L_{i1} = s_{0} f/2\quad {\text{for}}\quad i \ge a_{m} ,L_{i+ 1,i} = s\quad {\text{for}}\quad i = 1, \ldots ,72,\quad {\text{and}}\quad L_{ij} = 0\,{\text{otherwise}}{.}$$

We used the population growth rate *λ*, defined by the maximum eigenvalue of **L**, as an indicator of the population growth rate.

We assumed that the sex of each individual in 1979 was randomly sampled by the 1:1 sex ratio, and its age was randomly sampled by the stable age structure that is given by the eigenvector of the Leslie matrix with the maximum eigenvalue. We assumed that the number of individuals at age 1 year in year *t* + 1, denoted by *N*_1,*t*+1_, is determined by the binomial distribution:

$$Pr\left[ {N_{1,t + 1} = x} \right] = \left( {\begin{array}{*{20}c} {N_{f} } \\ x \\ \end{array} } \right)\left( {s_{0} f} \right)^{x} \left[ {1 - \left( {s_{0} f} \right)} \right]^{{N_{f} - x}} ,$$

where *N*_*f*_ represents the number of adult females in year *t*. We assumed that no twins were born. We assumed that the probability that an individual with age x survived in the next year is s if *x* = 1 or s0 if *x* = 0. We also assumed that *C*_*t*_ individuals who died by bycatch were randomly chosen from any sex and age because the age of individuals caught by bycatch is rarely known. We do not know the sex of some individuals.

We assumed the following prior distributions for *N*_1997_, *f,* and *s*: *N*_1979_ $\in$ *U*(11, 80), *f* $\in$ *U*(1/14, 1/6) if at least one adult male existed in the population, *s*_0_ $\in$ *U*(0.1, 0.85); and *s* $\in$ *U*(0.8, 0.97), where *U*(*a*, *b*) is the uniform random variable between *a* and *b*. These probabilities were constant for each simulation trial from 1997 to 2019. We selected the set of parameters with the population growth rate (λ) obtained when the maximum eigenvalue of the Leslie matrix was between 0.96 and 1.01.

We rejected trials that did not satisfy the following summary statistics: *N*_1997_ ≥ 11 (intensive survey in 1997), *N*_*t*_ ≥ 3 during 2004–2017 (monitoring), and *N*_2019_ ≤ 1 (“local extinction”). We obtained the prior distributions of *N*_1997_, *f*, *s*_0_, *s*, and *N*_2004_, and of the > 130,000 trials in the prior distribution with natural population growth rates λ of 96.1–98.8%, 99.3% were rejected. For 95% of the 1000 adopted trials, *N*_1979_ ranged from 14 to 58. If λ > 98%, *N*_1997_ was ≤ 45 for the adopted trials (Extended Data Fig. 7. Even if all the stranding deaths were due to anthropogenic factors, such as the release of dugongs after bycatch or boat strike, the range of *N*_1997_ changed to < 56 if λ > 98%, with only a slight upward shift, but positive natural growth rate (or λ > 1) was again very unlikely (0.3%) among the adopted trials.

### Population viability analysis to assess the impact of bycatch on the extinction risk

We re-evaluated the extinction risk with and without bycatch using the 1000 parameter sets of *N*_1979_, *f*, *s*_0_, and *s* that satisfied the summary statistics in the ABC and stochastic individual-based model, beginning from *N*_1979_ for the corresponding parameters. For each parameter set, 100 trials were conducted for each scenario to compare the extinction risks.

## Supplementary Information

Supplementary Information 1.

Supplementary Information 2.

## Data Availability

All data in this study are included in this article and its supplementary data files.

## References

[1] McMahon CR, Bester MN, Hindell MA, Brook BW, Bradshaw CJ (2009). Shifting trends: detecting environmentally mediated regulation in long-lived marine vertebrates using time-series data. Oecologia.

[2] Karczmarski L, Huang SL, Chan SC (2017). Threshold of long-term survival of a coastal delphinid in anthropogenically degraded environment: Indo-Pacific humpback dolphins in Pearl River Delta. Sci. Rep..

[3] Franklin, I. R. Evolutionary change in small populations. In *Conservation Biology: An Evolutionary-Ecological Perspective* (eds Soulé, M. E. & Wilcox, B. A.) 135–149 (Sinauer Associates Inc., 1980)

[4] Marsh, H. & Sobtzick, S. *Dugong dugon* (amended version of 2015 assessment). in *The IUCN Red List of Threatened Species.* e.T6909A160756767 (International Union for Conservation of Nature, 2019).

[5] Li B, Huang D, Zeng G, Cheng M, Lai C, Zhang C, Xu P, Xue W, Hu X (2019). Dugongs under threat. Science.

[6] Marsh, H. The life history, pattern of breeding, and population dynamics of the dugong. In *Population Biology of the Florida Manatee,* (eds O’Shea, T. J., Ackerman, B. & Percival, H. F.) 75–83 (1995).

[7] Marsh H, Kwan D (2008). Temporal variability in the life history and reproductive biology of female dugongs in Torres Strait: The likely role of sea grass dieback. Contin. Shelf Res..

[8] Marsh, H., O'Shea, T. J. & Reynolds, J. E. (eds) *Ecology and Conservation of the Sirenia: Dugongs and Manatees*. 521p. (Conservation Biology, Cambridge University Press, 2011).

[9] Hines, E. M. Dugongs in Asia. in *Sirenian Conservation: Issues and Strategies in Developing Countries,* (eds Hines, E. M., Reynolds, J. E., Aragones, L. V., Mignucci-Giannoni, A. A. & Marmontel, M.) 58–76 (University Press of Florida, 2012).

[10] Ikeda, K. & Mukai, H. Dugongs in Japan. in *Sirenian Conservation: Issues and Strategies in Developing Countries,* (eds Hines, E. M., Reynolds, J. E., Aragones, L. V., Mignucci-Giannoni, A. A. & Marmontel, M.) 77–83 (University Press of Florida, 2012).

[11] Brownell Jr., R.L., Kasuya, T. & Marsh, H. *Dugong dugon* (Nansei subpopulation). in *The IUCN Red List of Threatened Species 2019*: e.T157011948A157011982 (International Union for Conservation of Nature, 2019).

[12] Shirakihara M, Yoshida H, Yokochi H, Ogawa H, Hosokawa T, Higashi N, Kasuya T (2007). Current status and conservation needs of dugongs in southern Japan. Mar. Mam. Sci..

[13] Toyama, M. Decline of dugong populations around the Ryukyu Archipelago: temporal pattern since the 19th century and its causal factors as inferred from the annual prefectural statistics of fisheries in Okinawa and a survey for relevant information appearing in old local newspapers. *Aqua (Kaiyo-to-Seibutsu)* **37**, 351–356 (2015); **(in Japanese with English abstract)**.

[14] Uni Y (2003). Harvest statistics of Dugong dugon in Okinawa Prefecture. Ajima Bull. Nago Museum.

[15] Toyama, M. Overexploitation and extinction history of dugongs. in *Environmental History of Islands, Ocean and Forest,* (eds Tajima, Y. & Ankei, Y.) 173–194 (Bun-ichi Co., Ltd., 2011); **(in Japanese)**.

[16] Kakuma S (2007). Destructive fisheries and aquaculture in Southeast Asia-Towards balancing coral reef eco-system and fishery. J. Reg. Fish..

[17] Yamamuro M, Aketa K, Uchida S (2004). Carbon and nitrogen stable isotope ratios of the tissues and gut contents of a dugong from the temperate coast of Japan. Mamm. Study.

[18] Sirenia Specialist Group. *A Research Plan for the Japanese Dugong Sub-Population*. Expert Workshop held at Toba Aquarium, 21p (2019).

[19] Okinawa Defense Bureau. https://www.mod.go.jp/rdb/okinawa/07oshirase/chotatsu/kankyoukansiiinkai/kankyoukansiiinkai33/R03no33Siryo08.pdf (2020)

[20] Ministry of the Environment, Broad-area Survey of Dugongs in FY 2020. **(in Japanese)** https://www.env.go.jp/nature/kisho/r2_jyugon.pdf (2021)

[21] Nature Conservation Division, Environment Department, Okinawa Prefecture, Report on Dugong Protection Project in FY2020 **(in Japanese)** https://www.pref.okinawa.jp/site/kankyo/shizen/documents/r3honpen.pdf (2021).

[22] Sheppard JK, Preen AR, Marsh H, Lawer IR, Whiting SD, Jones RE (2006). Movement heterogeneity of dugongs, *Dugong dugon* (Müller), over large spatial scales. J. Exp. Mar. Biol. Ecol..

[23] Hobbs J-PA, Frisch AJ, Hender J, Gilligan JJ (2007). Long-Distance Oceanic Movement of a Solitary Dugong (*Dugong dugon*) to the Cocos (Keeling) Islands. Aquat. Mamm..

[24] Harting AL, Baker JD, Johanos TC (2017). Estimating population size for Hawaiian monk seals using haulout data. J. Wild. Mgmt..

[25] Naha Defense Facilities Administration Agency, Report of Field Survey offshore Schwab (Part 1) **(in Japanese)** (1997).

[26] Kasuya T, Shirakihara M, Yoshida H, Ogawa H, Yokochi H, Uchida S, Shirakihara K (1999). Japanese dugongs, their current status and conservation measures required. Annual Report of Pro Natura Fund.

[27] Dugong Network Okinawa. Survey on dugong inhabitant and seagrass in the eastern Okinawa Island and application to environmental education **(in Japanese)**. *WWF Seminar in Nansei Islands*, 15p (1999).

[28] Defense Facilities Administration Agency, Preliminary Study of the Habitat of Dugongs **(in Japanese)**, 65p. (2001).

[29] Ministry of the Environment, Broad-area Survey of Dugongs and Seagrass Meadows in 2001–2005 **(in Japanese)** (2006).

[30] Okinawa Defense Bureau, Follow-up Investigation Report in FY2019 **(in Japanese)**. https://www.mod.go.jp/rdb/okinawa/07oshirase/chotatsu/jigochousa01/Jigochousa01.html (2020)

[31] Okinawa Defense Bureau, Final Environmental Impact Assessment of Futenma Replacement Facility Construction Project **(in Japanese)** https://www.mod.go.jp/rdb/okinawa/07oshirase/chotatsu/hyoukasyohosei/hyoukasyohosei.html (2012)

[32] Csilléry KM, Blum GB, Gaggiotti OE, François O (2010). Approximate Bayesian Computation (ABC) in practice. Trends Ecol. Evol..

[33] Matsuda H, Yamamura O, Kitakado T, Kobayashi Y, Kobayashi M, Hattori K, Kato H (2015). Beyond dichotomy in the protection and management of marine mammals in Japan. Therya.

