# Supplementary material for: Trajectory to local extinction of an isolated dugong population near Okinawa Island, Japan
Source: Sci Rep. 2022 Apr 12;12:6151. doi: 10.1038/s41598-022-09992-2 (PMC9005736; doi:10.1038/s41598-022-09992-2)
Supplement: Supplementary file 1 — Supplementary Information 1. [file 41598_2022_9992_MOESM1_ESM.pdf]

Trajectory to local extinction of an isolated dugong population near Okinawa Island, Japan

Hajime Kayanne, Takeshi Hara, Nobuaki Arai, Hiroya Yamano, Hiroyuki Matsuda

Supplementary Information

This document contains:
Extended Data Figures 1 to 8.
Supplementary Table 1.

As separate Excel file:
Supplementary Data 1. Identification of dugong individuals along Okinawa Island coast.

a

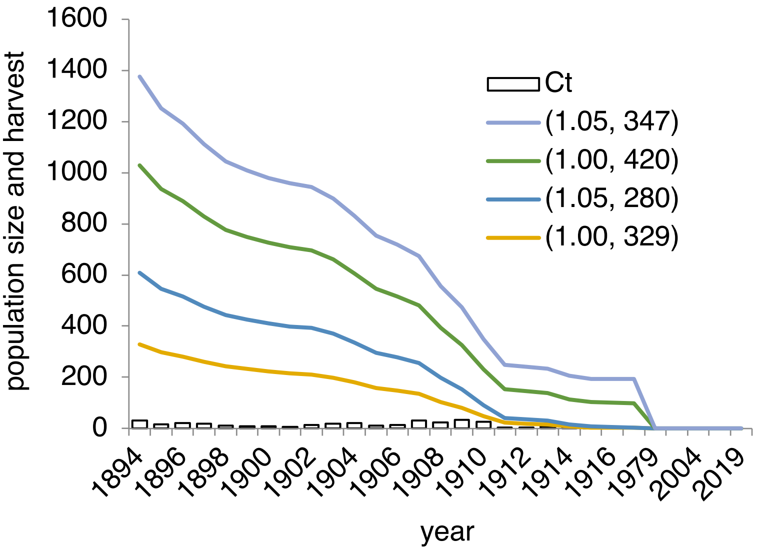

b

year	Okinawa	Miyako	Yaeyama	total
1893	-	-	2	2
1894	-	7	24	31
1895	2	-	14	16
1896	2	-	19	21
1897	7	2	9	18
1898	3	-	10	13
1899	3	1	5	9
1900	-	-	7	7
1901	1	-	4	5
1902	8	-	5	13
1903	15	-	4	19
1904	3	6	12	21
1905	3	3	5	11
1906	6	1	6	13
1907	-	2	29	31
1908	-	-	23	23
1909	-	-	33	33
1910	1	1	24	26
1911	1	-	2	3
1912	1	-	2	3
1913	1	2	5	8
1914	1	-	3	4
1915	1	-	-	1
1916	1	-	-	1
total	60	25	247	332

Extended Data Fig. 1: Change in dugong population sizes between 1893 and 1916. a, Estimates of the numbers of dugongs near Ryukyu Islands. The four lines show the population size (N_t) with scenarios (λ, N_{1894}) based on harvest records (C_t). **b,** Table of harvest records reconstructed from fishery statistics[1,2].

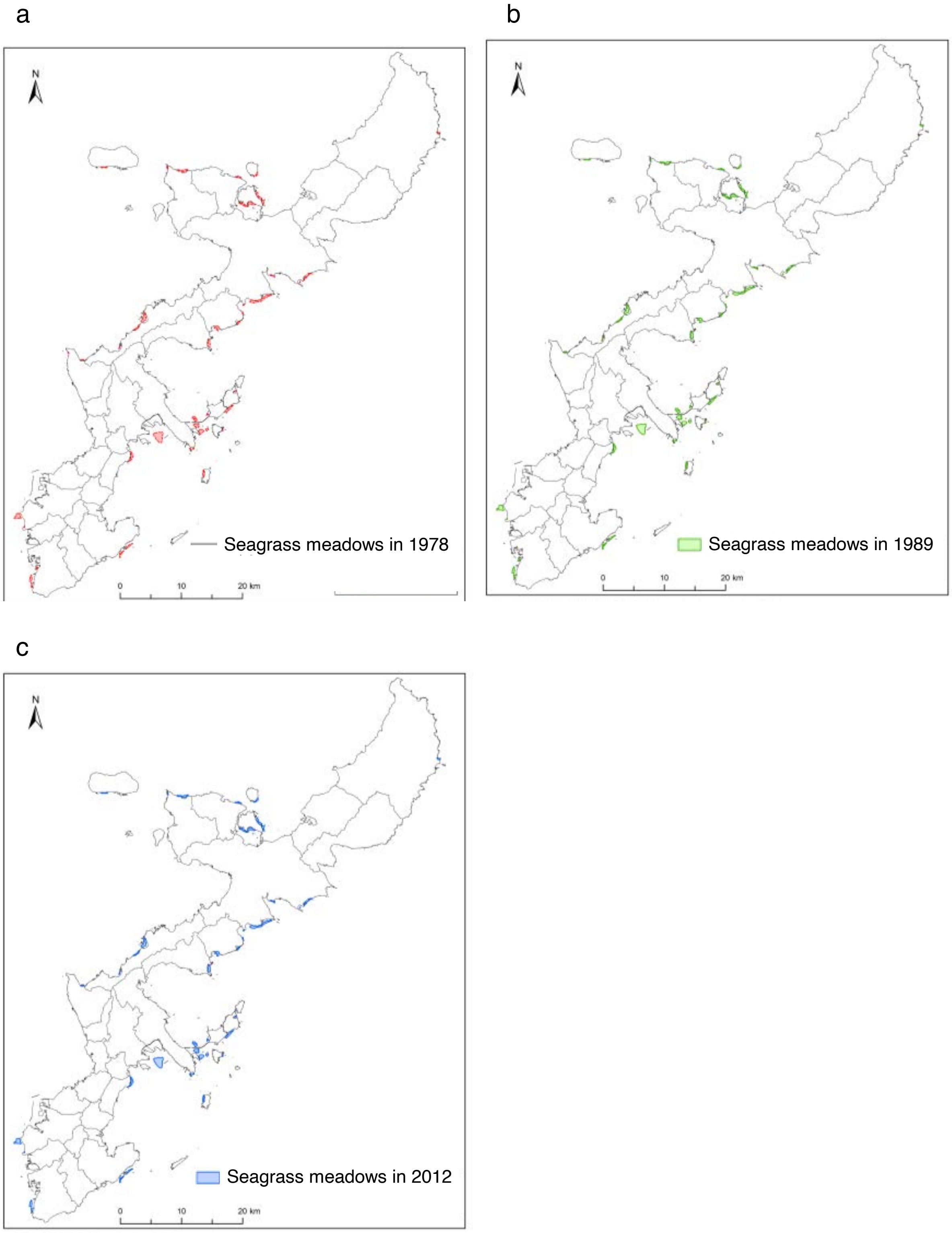

Extended Data Fig. 2: Change in areas of seagrass meadows. area in 1978 (**a**); in 1989 (**b**); and in 2012 (**c**). Data source: for 1978 (ref. [3]); for 1989 (ref. [4]); for 2012 (ref. [5]). Maps were generated by ArcGIS Desktop, ArcMAP10.7.1, https://www.esri.com/ja-jp/arcgis/products/arcgis-desktop/resources

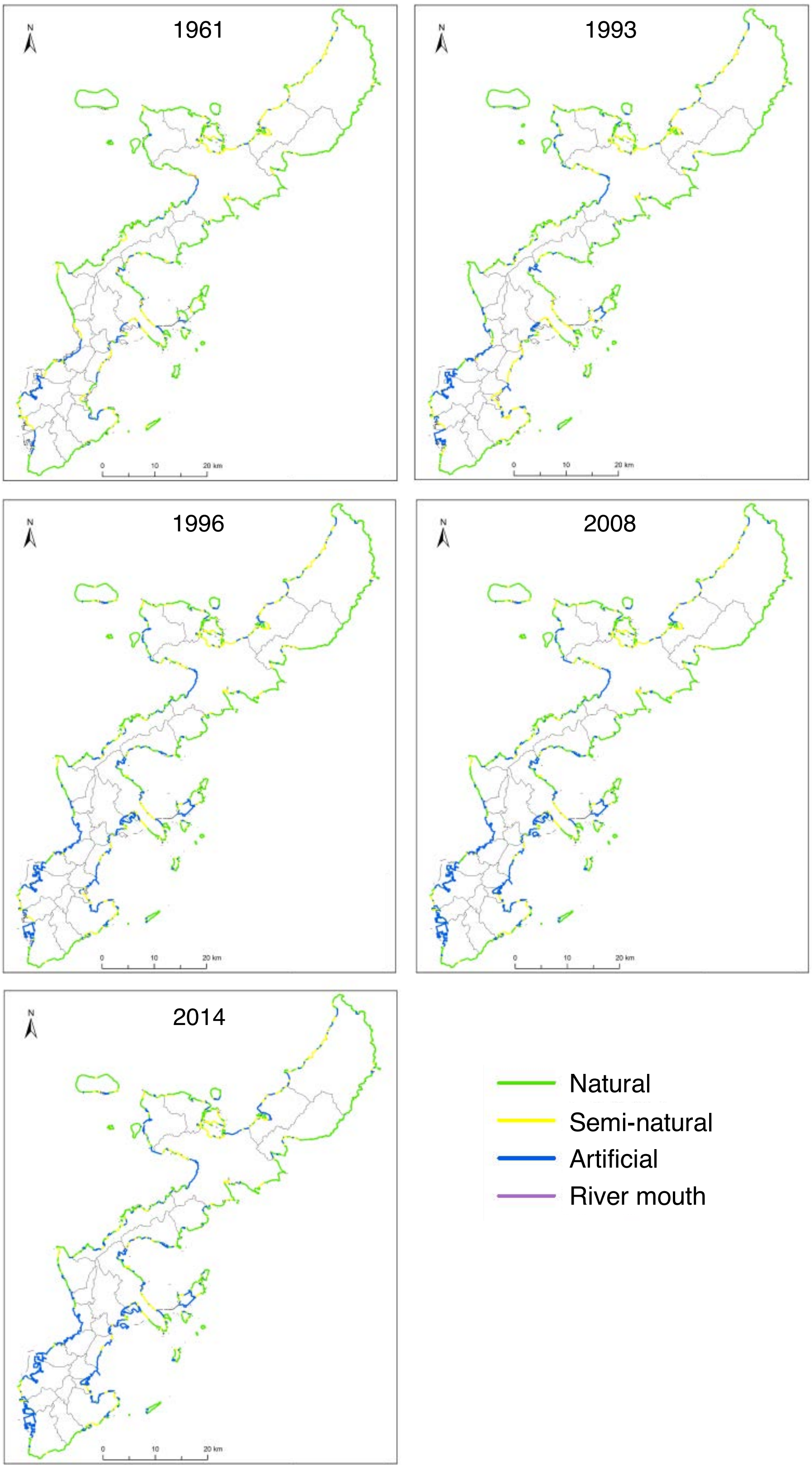

Extended Data Fig. 3: Change in the coastline around Okinawa Island. Natural (green), semi-natural (yellow), artificial (blue), and river mouth (purple). Data source: for 1961 and 2008 (ref. [5]); for 1993 (ref. [6]); for 1996 (ref. [7]). The figure for 2014 was prepared based on topographic maps and aerial photographs of the Geospatial Information Authority of Japan. Maps were generated by ArcGIS Desktop, ArcMAP10.7.1, https://www.esri.com/ja-jp/arcgis/products/arcgis-desktop/resources

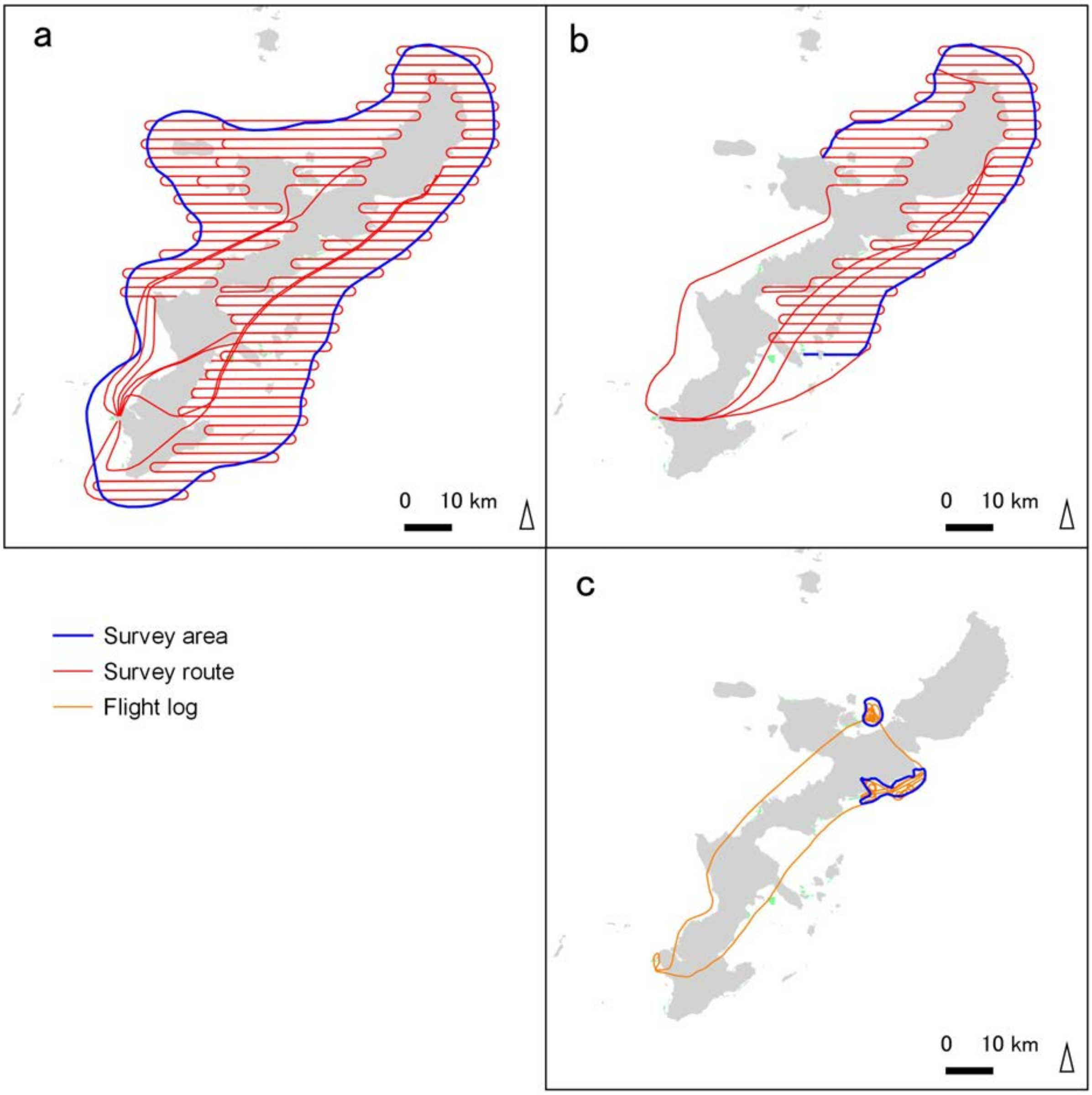

Extended Data Fig. 4: Representative survey tracks of the aircraft[8, 9]. a, Once a month from 2007 to June 2009. **b,** Four times a year since 2009. **c,** Once a week by helicopter since 2014. Maps were created by QGIS, ver. 3.10.6, https://www.qgis.org/ja/site/forusers/download.html

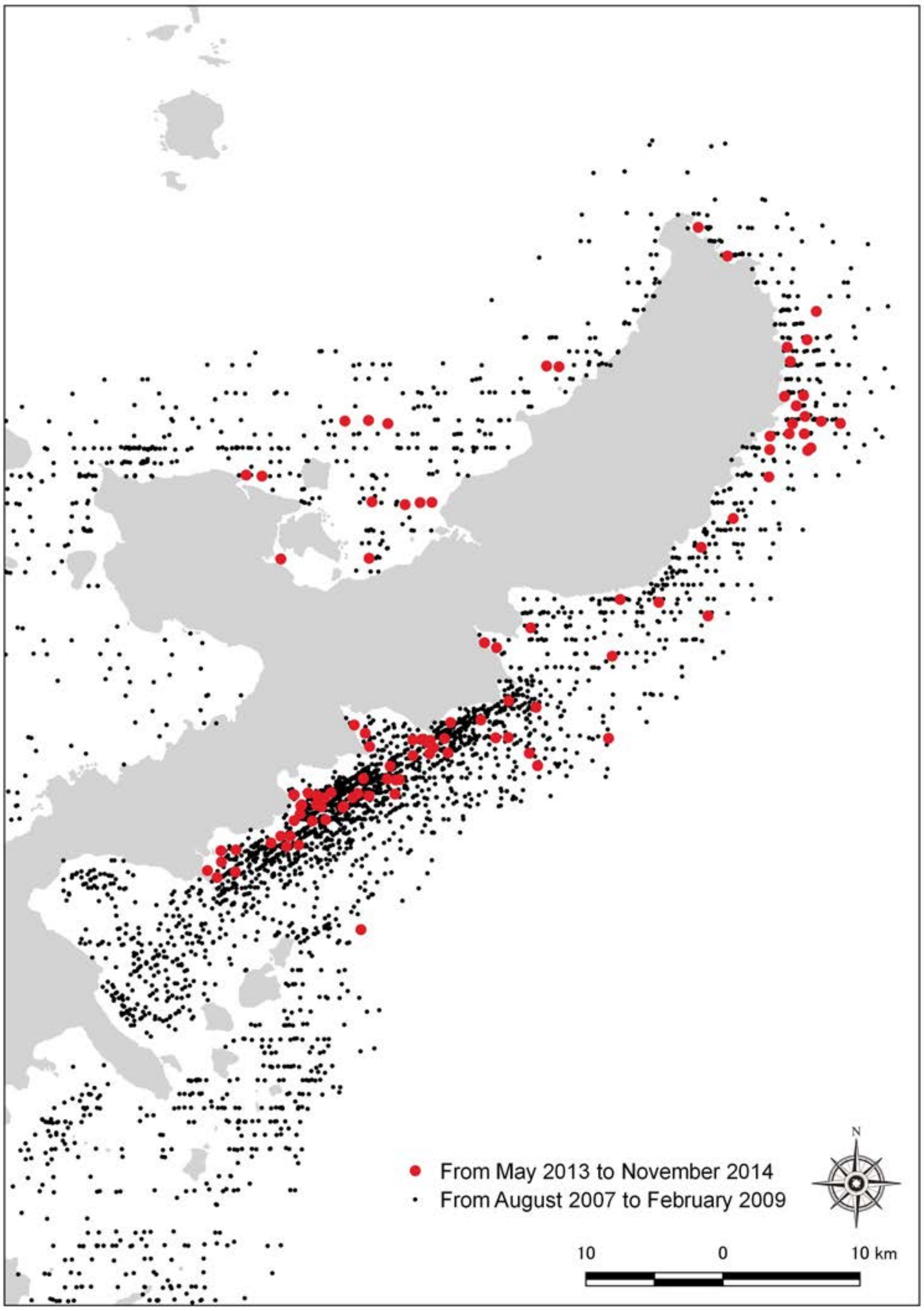

Extended Data Fig. 5: Records of sea turtle observations during the dugong surveys by aircraft and helicopter. Data source: from 2013 to 2014 (red circles: Okinawa Defense Bureau, 2014, 2015, Schwab Aquatic Animals etc. Survey Report); from 2007 to 2009 (black dots: ref. [9]). Maps were created by QGIS, ver. 3.10.6, https://www.qgis.org/ja/site/forusers/download.html

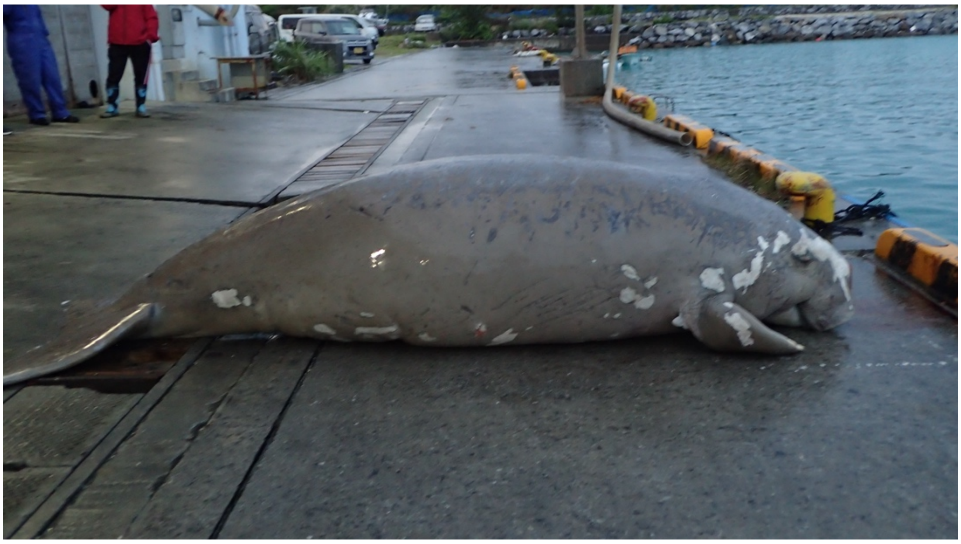

Extended Data Fig. 6: Dead body of individual B[10]. Found on 18 March 2019 at Unten fishing port, east coast of Okinawa.

a b

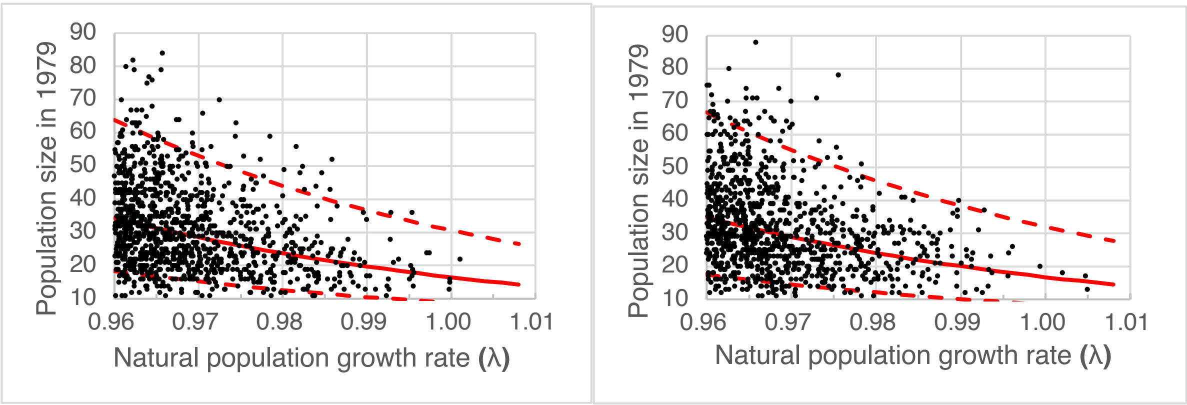

Extended Data Fig. 7: Relationship between the intrinsic rate of population increase (λ) and population size in 1979 (N_{1979}) for trials (dots) that satisfy the summary statistics. **a,** Calculation based only on dugong deaths by bycatch. **b,** Calculation assuming that all the reported deaths were caused by anthropogenic factors. The solid and dashed lines indicate the median of $N_{1979}\lambda^{18}$ and its 95% confidence interval, respectively.

a

b

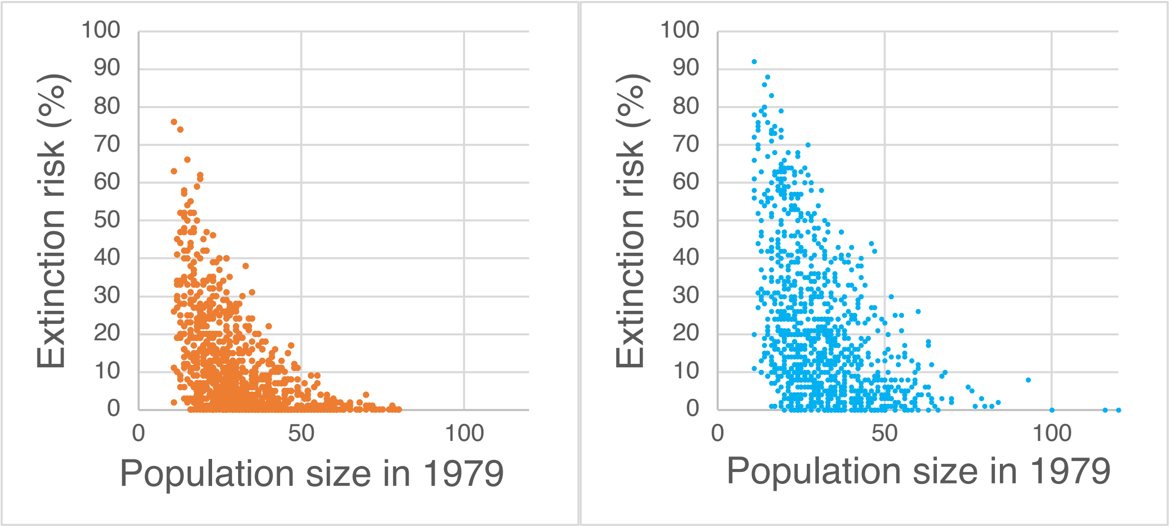

Extended Data Fig. 8: Extinction risk from 1000 sets of the adopted population size in 1979 with and without all reported deaths (panels a and b, respectively). From the 1000 pairs of adopted N_{1979} and λ in Fig. 7b, we calculated the extinction risk for each set. The extinction risk reduced with increasing N_{1979}.

Supplementary Table 1. Record of caught or drifted dugong individuals.
Based on (ref. [11]), with addition and revision from the source of newspaper accounts and the recent one of N24.

No.	Indv no.	date (year/month/ day)	site	sex	body length (cm)	body weight (kg)	assumed age	condition	Ref.	Newspaper articles	
N1		1960/12/6	Kise, Kasari, Amami Oshima Is.	M		270	adult	Hunted.	Ref. 12	Nankai-Nich-Nichi	Driven into the shallow, captured and sold to local people for meat,
N2		1965/10/25	Miyako: Sarahama, Irabu-jima Is (RS) or west coast of Ikema-jima Is, (OT)	M	203	349	adult	Caught by drive-hunting.		N2-1 RS N2-2 OT	Drive-hunted ashore by two fishermen, then killed and sold for a specimen. For the first time in 50 years in Miyako Island. Site of catch was differed between N2-1 and N2-2. Described as female in N2-2, but as make in the later examination.
N3		1967/5/9	Kanokawa Bay, Iriomote Is,	F, M		ca.300 (F) ca.180 (M)	adult calf	Stranded and caught ashore.		N3 OT	Two individuals were stranded and clubbed to death by two fishermen, then sold at market. It was the third times fot the fisherman to catch dugongs.
N4		1979/1/18	Kayo, Okinawa	F	159	95	2 to 3 years	Cautht in gill net		N4-1-1,2,3,4,5,6,7,8,9,10,11,12,13 RS N4-2-1,2,3,4,5,6,7,8,9,10,11,12 OT	Age 2-3 years: caught in gill net alive. Disputed for release or breeding, but died in 33 days later. Specimen was preserved in prefecture museum.
N5		1982/3/27	Kanna, Ginoza, Okinawa	M	251	267	adult	Stranding ashore		N5 OT	Many scratches on body, Preserved in Okinawa Marine Park Aquarium.
N6		1984/4/24	Gushikawa, Okinawa	M	262	—	adult	Dead body drifted ashore		N6-1 RS N6-2 OT	Preserved in Okinawa Marine Park Aquarium.
N7		1987/5/10	Uchipanari-jima, Iriomote Is.	—	ca.200	—	adult	Dead body on shore	Ref. 13	N7 RS	Caudal part was skeletonized.
N8		1988/1/4	Fusozaki, Sasiki, Nakagusuku Bay, Okinawa	F	251	290	adult	Dead body drifted ashore		N8-1 RS N8-2 OT	Preserved in Univ. Ryukyus.
N9		1988/1/14	Kanna Kochidani, Ginoza, Okinawa	M	187	146	calf	Dead body drifted ashore		N9-1 RS N9-2 OT N9-3 OT	Prematured. Examined by Okinawa Marine Park Aquarium. Agglomerated fishlines were found from stomach.
N10		1990/5/16	Kayo, Nago, Okinawa	M	117	39	3 months	Caught in gill net	Ref. 14,15 D1 in ref.16	N10-1 RS N10-2 OT	Three months old with naval string attatched. Caught in gill net alive, but soon died.
N11		1992/5/9	Kin, Okinawa	M	200	173	calf	Cautht in fixed net, bred in aquarium but dead		N11-1 RS N11-2 OT	Straying into fixed net. Captured and bred in aquarimu, but not reported afterwards. Dead shortly.
N12		1992/5/9	Kin, Okinawa	F	266	374	adult	Caught in fixed net	D2 in ref.16	N12-N16 OT	This dugong was captured with N11 but not reported in N11.
N13		1993/12/4	Kin, Okinawa	M	196	—	calf	Caught in fixed net, released		N12-N16 OT	Only described in review article in N12-N16
N14		1995/12/28	Abu, Nago, Okinawa	F	296	560	adult , pregnant	Caught in fixed net	Ref. 17	N14 RS N12-N16 OT	Probably pregnant. Examined by Okinawa Marine Park Aquarium.
N15		1996/1/15	Kouri-jima, Nakijin, Okinawa	M	ca.300	—	adult	Caught in fixed net	D3 in ref.16	N12-N16 OT	Described in review article in N12-N16, and exemined in Ref.4
N16		1997/1/22	Kanna, Ginoza, Okinawa	M	267	—	adult?	Caught in fixed net, released		N16 RS N12-N16 OT	
N17	J	1998/11/13	Henza-jima, Yonashiro, Okinawa	M	110	31.7	2 months	Caught in gill net	Ref. 14.15 D4 in ref.16	N17-1RS N17-2 OT	Two months old. Struggled in gill net, then died.
N18	N	1999/4/1	Miyagi, Higashi-son, Okinawa	—	ca.300	—	adult	Dead body drifted ashore		N18 OT	First time in Higashi-son.
N19	H	2000/4/5	Ketomobara, Ginoza, Okinawa	M	255	250	adult	Dead body drifted ashore	D5 in ref.16	N19-1 RS N19-2 OT	Examined and preserved in Okinawa Marine Park Museum.
N20	E	2000/8/27	Offshore Sesoko, Motobu, Okinawa	F	298	395	adult	Dead body drifted offshore	D6 in ref.16	N20-1,2 RS N20-3,4 OT	Dead body drifetd nearshore, which had been observed in June 2000.
N21	I	2000/11/13	Kanna, Ginoza, Okinawa	F	218	243	calf	Caught in fixed net		N21-1 RS N21-2 OT	Call for measures to prevent by-catch.
N22		2002/10/9	Ushibuka, Kumamoto	M	230	158	calf	Dead body drifted ashore	Ref. 15,17 18	N22-1,2 Asahi	Caught in fixed net then escaped in 2002/10/4, Dead body was reported to be found on 2002/9/9 in Ref.4 (probably 2002/10/9).
N23	K	2004/4/26	Hija River mouth, Yomitan-son, Okinawa	—	200-300	—	adult	Caught in fixed net, released		N23-1,2,3 RS N23-,4,5 OT	Caught in fixed net, then rescued with much efforts by fishermen and released.
N24	B	2019/3/18	Unten fishing port, Okinawa	F	290	480.3	adult	Dead body drifted ashore	Ref.19	N24-1-1,2,3,4,5,6 RS N24-2-1,2,3,4,5,6,7,8 OT	Individual B
			Yellow: found outside Okinawa Island.				Green: calf	Red: Caught in gill/fixed nets or hunted. Blue: Dead body drifted ashore. Green: Caught then released or bred.		RS: Ryukyu Shimpo. OT: Okinawa Times	

SI References

1. Uni, Y. Harvest statistics of Dugong dugon in Okinawa Prefecture (**in Japanese**). *Ajima, Bulletin of Nago Museum* **11** (2003).
2. Toyama, M. Overexploitation and extinction history of dugongs (**in Japanese**). in *Environmental History of Islands, Ocean and Forest,* (eds Tajima, Y. & Ankei, Y.) 173–194 (Bun-ichi Co., Ltd., 2011).
3. Environment Agency, The Second Basic Survey for Nature Conservation (**in Japanese**) (1978).
4. Environment Agency, The Fourth Basic Survey for Nature Conservation (**in Japanese**) (1989).
5. Okinawa Prefecture, Integrated Report on the Project for Establishing Guidelines for Natural Environment Restoration (tentative title) (**in Japanese**) https://www.pref.okinawa.jp/site/kankyo/saisei/documents/saisei03.pdf (2016).
6. Ministry of the Environment, The 4th Basic Survey for Nature Conservation: Coastal Survey (**in Japanese**) http://www.biodic.go.jp/reports2/4th/kaigan/4_kaigan.pdf (1994).
7. Ministry of the Environment, The 5th Basic Survey for Nature Conservation: Coastal Survey (**in Japanese**) https://www.biodic.go.jp/reports/umibe/umibe.pdf (1998).
8. Okinawa Defense Bureau, Follow-up Investigation Report in FY2019 (**in Japanese**) https://www.mod.go.jp/rdb/okinawa/07oshirase/chotatsu/jigochousa01/Jigochousa01.html (2020).
9. Okinawa Defense Bureau, Final Environmental Impact Assessment of Futenma Replacement Facility Construction Project (**in Japanese**) https://www.mod.go.jp/rdb/okinawa/07oshirase/chotatsu/hyoukasyohosei/hyoukasyohosei.html (2012).
10. Okinawa Defense Bureau. https://www.mod.go.jp/rdb/okinawa/07oshirase/chotatsu/kankyoukansiiinkai/kankyoukansiiinkai19/H30no19Siryo05.pdf (**in Japanese**) (2019).
11. Ministry of the Environment, Broad-area Survey of Dugongs and Seagrass Meadows in 2001-2005 (**in Japanese**) (2006).
12. Nakahara,K. The story of Dugong (**in Japanese**). *Chirimos* **4**, 27-33 (1993).
13. Ohtaishi,N. Dugong biology and population management vision (**in Japanese with English abstract**). *Aqua.(Kaiyo-to-Seibutsu)* **37**, 339-344 (2015).

14. Ministry of the Environment, Broad-area Survey of Dugongs and Seagrass Meadows in FY 2001 (**in Japanese**) (2002).
15. Ministry of the Environment, Broad-area Survey of Dugongs and Seagrass Meadows in FY 2002 (**in Japanese**) (2003).
16. Aketa,K. *Study on Feeding Characteristics and Digestive Function of Sirenians* (**in Japanese**). Doctor Thesis in the Faculty of bioresources, Mie University (2003).
17. Ministry of the Environment, Broad-area Survey of Dugongs and Seagrass Meadows in FY 2003 (**in Japanese**) (2004).
18. Yamamuro, M., Aketa, K. & Uchida, S. Carbon and nitrogen stable isotope ratios of the tissues and gut contents of a dugong from the temperate coast of Japan. *Mamm. Study* **29**, 179–183 (2004).
19. Okinawa Prefectural Environment Department Nature Conservation Division, Dugong Conservation Measures Project Report in FY 2019 (**in Japanese**) (2020).
